# Functional connectivity alteration after real-time fMRI motor imagery training through self-regulation of activities of the right premotor cortex

**DOI:** 10.1186/s12868-015-0167-1

**Published:** 2015-05-01

**Authors:** Fufang Xie, Lele Xu, Zhiying Long, Li Yao, Xia Wu

**Affiliations:** College of Information Science and Technology, Beijing Normal University, No. 19 Xin Jie Kou Wai Da Jie, 100875 Beijing, China; State Key Laboratory of Cognitive Neuroscience and Learning & IDG/McGovern Institute for Brain Research, Beijing Normal University, 100875 Beijing, China; Center for Collaboration and Innovation in Brain and Learning Sciences, Beijing Normal University, 100875 Beijing, China; State Key Laboratories of Transducer Technology, Chinese Academy of Sciences, Shanghai, 200050 China

**Keywords:** Motor imagery training, Real-time functional magnetic resonance imaging, Premotor area, Functional connectivity, Graph theory

## Abstract

**Background:**

Real-time functional magnetic resonance imaging technology (real-time fMRI) is a novel method that can be used to investigate motor imagery training, it has attracted increasing attention in recent years, due to its ability to facilitate subjects in regulating the activities of specific brain regions to influence their behaviors. Lots of researchers have demonstrated that the right premotor area play critical roles during real-time fMRI motor imagery training. Thus, it has been hypothesized that modulating the activity of right premotor area may result in an alteration of the functional connectivity between the premotor area and other motor-related regions.

**Results:**

The results indicated that the functional connectivity between the bilateral premotor area and right posterior parietal lobe significantly decreased during the imagination task.

**Conclusions:**

This finding is new evidence that real-time fMRI is effective and can provide a theoretical guidance for the alteration of the motor function of brain regions associated with motor imagery training.

**Electronic supplementary material:**

The online version of this article (doi:10.1186/s12868-015-0167-1) contains supplementary material, which is available to authorized users.

## Background

Motor training, as an effective means of motor skills learning and motor function rehabilitation, has received widespread attention in the fields of neuroscience, cognitive science and medical science [1,2]. In particular, motor imagery training, which is an important aspect of motor training, has been found to be effective for patients who had completely lost motor execution abilities [2]. Real-time functional magnetic resonance imaging (real-time fMRI) is a novel technique that can be used to investigate motor imagery training. It enables subjects to adjust the activities of brain regions, such as emotions and behaviors, to influence their performance [3,4].

A large number of researchers have investigated the regulations of specific brain regions while performing real-time fMRI motor imagery training [5-8]. Some previous studies showed that the activity of the orbitofrontal cortex, which is associated with contamination anxiety and somatosensory information, could be used by subjects to modulate through real-time fMRI [5,6]. Furthermore, several other studies have demonstrated that the activity of the PMA is alterable during real-time fMRI training for both healthy participants [1,9-11] and stroke patients [10]. Sitaram et al. demonstrated a significantly increasing blood oxygenation level in the premotor cortex during feedback training [10]. Moreover, the prominent role of the rPMA during the early stages of skill learning, including spatial processing and sequence storage, has been investigated in some previous studies [12-14]. Zhang et al. determined the key role of the rPMA during motor imagery of the right hand and found a consistent correlation between the activity of the rPMA and motor performance of the right hand during a 2-week offline motor imagery training of the fingers of the right hand [15]. In general, the above studies suggested that regulating the activity of the rPMA may be effective for motor performance improvement. However, whether rPMA regulation can alter the motor function interaction between the PMA and other motor-related brain regions is still unknown.

Functional connectivity between brain regions should be considered an important physiological measure for real-time fMRI motor imagery training [3,16,17]. Several studies have demonstrated that real-time fMRI motor imagery training can result in an alteration of the functional connectivity between various brain regions. Functional connectivity during real-time fMRI motor imagery training for regulating activities in the insular [18,19], anterior cingulate cortex [20], right inferior frontal gyrus [21], visual cortex [22] and motor cortex [23] was found to be different from that during training with sham feedback and during training without feedback. Based on the granger causality model, Zhao et al. demonstrated that the interaction of the target region with other related regions is significantly altered through modulation of the activity of the dorsal PMA [11]. Nevertheless, there are still few neuroimaging studies on the functional interactions between PMA and other motor related regions by rPMA regulation.

The present study investigated the alteration of the functional connectivity between the PMA and other brain regions engaged in an imagination task after regulating the activities in the rPMA. There are many methods for measuring functional connectivity, such as correlation [24], coherence [25], beta serial correlation [26] and graph theory [27]. While correlation, coherence and beta serial correlation, which captures the pair-wise information between only two brain regions, can identify two brain regions that are functionally connected, they are unable to completely characterize the joint interactions between multiple brain regions [28]. Graph theory is a mathematical method that can be used to assess the properties of systems that can be modeled as sets of nodes (i.e., brain regions) and edges (i.e., functional connections) [29]. It can be used to quantitatively describe local and overall features of the network and has attracted increasing attention in the neuroscience community in recent years [30-33]. However, there are some potential problems regarding the interpretation of results obtained using this method, particularly for stimulus driven tasks. The graph theory method was improved by removing the stimulus-locked response to investigate the intrinsic task-related functional connectivity of critical areas [34]. In the present study, the improved graph theory was used to calculate the functional connectivity between the PMA and the motor-related regions that play critical roles in motor imagery training. Based on the findings of previous studies, real-time fMRI motor imagery training can change the functional interaction between various brain regions, especially the target ROI and other regions [11,19-21,35-37]. Thus, it has been hypothesized that modulating the activity of the rPMA may alter the functional connectivity of the motor network engaged in imagination tasks, especially the functional connectivity between the PMA and other motor-related regions.

## Method

In this section, we will describe the experiments and data analysis in detail.

### Participants

The experiment was performed by the Institutional Review Board of the State Key Laboratory of Cognitive Neuroscience and Learning at Beijing Normal University. Participants with neurological disorders and psychiatric disorders were excluded. Twelve right hand-dominant subjects were recruited as the experimental group (mean age 23 ± 2.14 years, six males and six females); they were administered true neurofeedback training. Another twelve right hand-dominant subjects were recruited as the control group (mean age 23 ± 1.7 years, eight males and four females); they were administered a sham neurofeedback signal randomly selected from the feedback curves of the experimental group. All participants had normal neurological examinations and were right-handed according to the Edinburgh Handedness Inventory, which includes the Movement Imagery Questionnaire and Vividness of Movement Imagery Questionnaires [38,39]. These questionnaires assessed the participants understanding of kinesthetic imagery, and we asked them to employ this imagery strategy during the entire experimental procedure. All participants provided written consent, according to the guidelines of the MRI Center of Beijing Normal University, before undergoing the experimental sessions.

### Experimental procedures

The experimental procedure consisted of a pre-scan practice, pre-training scan, real-time fMRI neurofeedback training (true feedback for the experimental group and sham feedback for the control group), a post-training scan and a questionnaire interview conducted outside of the scanner.

#### Pre-scan practice

Pre-scan practice was performed to familiarize the participants with the finger tapping task. Outside of the scanner, all participants were instructed that each of the four fingers of their right hand from their index to little finger represented a single digit number: one, two, three, and four. Then, they were instructed to imagine tapping their 1, 2, 3 and 4 at 4 Hz for a 30-s period to learn the rhythm required in the following scan session. Then, they were instructed to follow the set sequence 4-2-3-1-3-4-2 at 4 Hz for a 30-s period. In the pre- and post-training scans and real-time fMRI neurofeedback training, participants imagined tapping fingers following the sequence by themselves based on the learned rhythm in the pre-scan practice. Both groups underwent the exact same practice. After finishing these exercises, the participants were prepared for the experimental session in the scanner.

#### Pre- and post-training scans

For the pre- and post-training scans, each participant was instructed to complete a motor imagery run in the MRI scanner. The 4.5-min run consisted of four 30-s task blocks of imagining the motor sequence alternated with five 30-s rest blocks. When PUSH was displayed on the screen, the participants were required to imagine the sequence 4-2-3-1-3-4-2 with their right hands at a self-paced rate of 4 Hz. When REST was displayed on the screen, the participants were instructed to relax. The type of task was visually presented on a semi-transparent screen at the end of the scanner bore, and the participants could view a reflection of the screen on a mirror mounted on the head coil. Cushions inside the head coil were used to reduce head movement.

#### Real-time fMRI neurofeedback training

The real-time fMRI system used in this experiment was set up by our research group to enable the on-line acquisition and on-line analysis of data and the on-line presentation of neurofeedback to the participants. When the data were continuously transmitted, the acquired images were preprocessed online to calculate the incremental linear de-trending of the time-series, detect 3D motion detection and perform spatial smoothing using a Gaussian kernel with full width at a half maximum (FWHM) of 8 mm. Then the data were analyzed with a cumulative general linear model using Turbo-Brain Voyage (TBV) software (Brain Innovation, Maastricht, The Netherland [40]). The dynamic statistical map, mean time courses of the target region of interest (ROI) and six head motion parameters were then exported to the TBV interface.

During the motor imagery run of pre-training, a subject-specific ROI with 5 × 6 × 1 voxels (approximately 15 × 18 × 5 mm^3^) was manually defined over the right dorsal PMA (dPMA), and a background area was separately derived according to the action map from the online General linear model (GLM) analysis by TBV software. The right dPMA was the target ROI to be updated by the participant. It was selected as rectangular zones centered on activation of the PMA. The mean location of the center of the selected target ROI across participants was x = 27, y = -1 and z = 58 based on the Montreal Neurological Institute template (MNI:[41]) coordinates. To cancel out global changes in blood-oxygen-level-dependent (BOLD), the background ROI was defined as a task-unrelated area one slice away from the target ROI. The feedback signals presented to the subject were calculated as the differences between the mean BOLD value in the target ROI and the mean BOLD value in the background ROI using the following equation, which was updated once per TR:

$$ \mathrm{curve}\ \mathrm{height} = {\left(\mathrm{B}\mathrm{O}\mathrm{L}{\mathrm{D}}_{\mathrm{training}}\hbox{-}\ \mathrm{B}\mathrm{O}\mathrm{L}{\mathrm{D}}_{\mathrm{rest}}\right)}_{\mathrm{targetROI}}\hbox{-} {\left(\mathrm{B}\mathrm{O}\mathrm{L}{\mathrm{D}}_{\mathrm{training}}\hbox{-}\ \mathrm{B}\mathrm{O}\mathrm{L}{\mathrm{D}}_{\mathrm{rest}}\right)}_{\mathrm{backgroundROI}} $$

**Figure 1:**
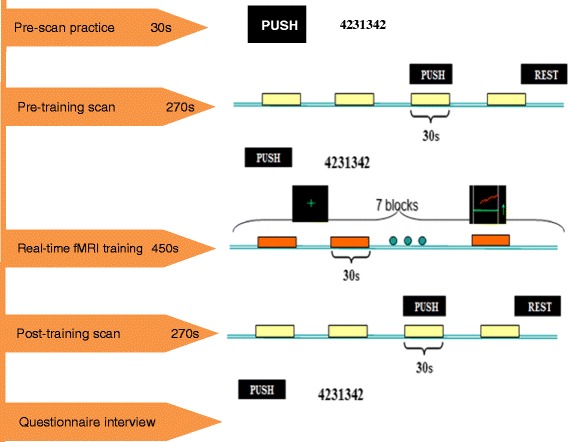
Outline of experimental procedures. In the pre-scan practice, subjects followed the set sequence 4-2-3-1-3-4-2 at 4 Hz for a 30-s period to learn the rhythm required in the following scan session. Each pre/post- training scan lasted 270 s, the 270 s run consisted of four 30-s task blocks of imagining the motor sequence alternated with five 30-s rest blocks. When PUSH was displayed on the screen, the participants were required to imagine the sequence 4-2-3-1-3-4-2 with their right hands at a self-paced rate of 4 Hz. When REST was displayed on the screen, the participants were instructed to relax. Each real-time fMRI training session lasted 450 s, during which time 30-s blocks of rest alternated with 30-s blocks of motor imagery with feedback, for a total of seven task blocks and eight rest blocks. During the rest blocks, a green “ + ” sign was presented on the screen; and during the task blocks, the green up arrow appeared on the screen along with the continually updated red curve. Questionnaire interview after scanning was to guarantee participants completed the motor imagery task well.

Following the pre-training run, the real-time fMRI training consisted of four 7.5-min sessions, each session included eight 30-s rest blocks alternated with seven 30-s task blocks with feedback, and the feedback was presented to the subject during the task block was a continuously updated curve (see Figure 1).

All participants received identical instructions regarding the strategies for increasing the activity they observed in the ROI when the green up arrow appeared on the screen. All participants received identical instructions to adjust their strategies of imaging the sequence 4-2-3-1-3-4-2 without finger movement and were informed that the most effective strategy would increase the height of the curve the most during imagery. The strategy instructions provided to all participants included varying the speed, strength and method of finger tapping. In addition, the participants were told that the feedback was inherently delayed in relation to cognitive events because of the delay in biologically inherent hemodynamics (3-5 s) and the computer preprocessing time (1-2 s) prior to scanning. When the green “ + ” sign was displayed on the screen, the subjects were instructed to relax and rest without recalling anything related to the regulation. The participants in the control group received the same experimental procedure and instruction as the experimental group, but they were supplied with a sham feedback signal randomly selected from the feedback curves of the experimental group.

#### Questionnaire interview after scanning

After finishing all runs in the scanner, the participants of both groups were asked to provide a qualitative description of the performance of the movement imagery during the entire fMRI experiment. The contents of the qualitative description were based on the Movement Imagery Questionnaire and included seven rating levels (1, very hard to feel; 2, hard to feel; 3, somewhat hard to feel; 4, neutral (not easy not hard); 5, somewhat easy to feel; 6, easy to feel; and 7, very easy to feel). Each participant should have rated the levels reliably, and no participants rated the performance of the movement imagery below 5, which indicated that they completed the motor imagery task well. This questionnaire interview could be used to guarantee that the participants did not really execute their fingers instead of just imagine it. We found that there was almost no activation in the left primary motor cortex (M1) during the motor imagery tasks compared with during executing motor tasks (seen in Additional file 1: Figure S1). Studies suggested that the left M1 was always activated in motor execution tasks such as rapid sequences of finger movement [42,43]. That is to say, the subjects did not really execute their fingers in the motor imagery tasks.

### Data acquisition

Brain scans were performed using a 3.0-T Siemens whole-body MRI scanner at the MRI Center of Beijing Normal University. For each participant, a single-shot T2*-weighted gradient-echo EPI sequence was used for functional imaging acquisition with the following parameters: TR = 2000 ms, TE = 40 ms, flip angle = 90°, acquisition matrix = 64*64, field of view (FOV) = 240 *240 mm; and slice thickness = 4 mm with inter-slice gap = 0.8 mm. Thirty-two axial slices parallel to the AC-PC line were obtained in an interleaved order to cover the entire cerebrum and cerebellum.

### Data analyses

#### Data preprocessing

The study was performed based on the processed data of our previous research. The functional images were first realigned, spatially normalized into standard stereotaxic space (EPI template provided by the MNI), re-sliced to 3 × 3 × 4 mm^3^ voxels, and smoothed using a Gaussian kernel with an 8*8*8 full-width at half maximum (FWHM) and SPM8 software (Statistical Parametric Mapping; http://www.fil.ion.ucl.ac.uk/spm). The first five images in each series were removed from further analysis. Using rest as the baseline, a general linear model (GLM) analysis was applied to each subject’s data processed by a high-frequency filter and global scaling with SPM8. Then, the task related t-contrast images were calculated using the t-statistic for each subject. A two-way repeated measures analysis of variance (ANOVA) using training (pre-test and post-test; within-subject; and fixed effect) and group (experimental and control; between-subjects) as the main factors was conducted to identify differences induced by training and group using SPSS 13.0 software (SPSS Inc., Chicago, IL, USA).

#### Definition of regions of interest (ROIs)

A brain functional work, as defined by graph theory, is composed of a number of nodes and a set of edges. Nodes can be denoted by the regions of interest (ROIs) and edges can be represented by functional connections between pairs of nodes. Considering the structural and functional alignments, ROIs were defined according to the results of group-level and individual-level analysis. The SMA, M1, PMA, cerebellum, putaman, PPL, and thalamus have been determined as the critical regions for motor sequence training [44,45]. Therefore, the present study paid close attention to these regions. However, the recruiting of M1 is still controversial in imagination tasks, and we did not observe any activities in the right M1 at the reduced threshold of p < 0.05 for MI tasks. Twelve ROIs (excluding the right M1) were finally focused on for the imagination task, according to the procedures of previous studies [27]. The brain regions with constant activation were selected as the ROIs [p < 0.05, cluster size > 41, FDR (false discovery rate) correction]. The ROIs were defined according to the procedures of previous study [8,11,27,46], for each ROI, the group spherical template was constructed using the spatial coordinates of the maximum activation in the group activation map as the center with a 10 mm radius; the MNI coordinates of these group ROIs are shown in Tables 1 and 2. The individual spherical template was constructed using the spatial coordinates of the maximum activation in the activation map for each individual as the center with a 6 mm radius. Then, for further functional connectivity analysis for each subject, the averaged time series from the normalized functional images was extracted from each individual ROI within which activation intensity reached a specified value (t > 2.33).

**Table 1:** **The coordinates and t-value of the peak voxel within group ROIs at pre-test and post-test for experimental group**

**Region(exp)**	**L/R BA**		**Pre-test**				**Post-test**			
			**Motor imagery task**				**Motor imagery task**			
			**MNI coordinate**			**T-score**	**MNI coordinate**			**T-score**
			**x**	**y**	**z**		**x**	**y**	**z**	
PMA	L	6	-27	-7	58	7.19	-27	-10	58	8.90
PMA	R	6	27	-7	58	9.13	27	-7	58	12.81
M1	L		-42	-16	58	6.44	-39	-16	58	7.52
PPL	L	7	-21	-61	58	4.62	-21	-61	58	5.42
PPL	R	7	36	-55	58	7.27	33	-58	58	5.96
SMA	L/R	6	-3	-1	58	11.62	-3	2	58	13.24
Putaman	L		-24	2	10	10.93	-24	-1	6	14.14
Putaman	R		24	2	10	9.76	24	2	6	12.20
Thalamus	L		-12	-19	6	11.04	-9	-22	2	14.10
Thalamus	R		15	-13	6	6.71	9	-19	-2	7.60
Cerebellum	L		-30	-58	-30	6.85	-24	-67	-26	7.96
Cerebellum	R		30	-58	-30	7.79	30	-58	-30	9.00

Note. MNI coordinates; Abbreviations; PMA-premotor area; M1-primary motor cortex; PPL-posterior parietal lobe; SMA-supplementary motor area; BA-Brodmann’s area.

**Table 2:** **The coordinates and t-value of the peak voxel within group ROIs at pre-test and post-test for control group**

**Region(ctr)**	**L/R BA**		**Pre-test**				**Post-test**			
			**Motor imagery task**				**Motor imagery task**			
			**MNI coordinate**			**T-score**	**MNI coordinate**			**T-score**
			**x**	**y**	**z**		**x**	**y**	**z**	
PMA	L	6	-27	-10	58	7.72	-27	-7	54	8.99
PMA	R	6	30	-10	58	4.5	27	-7	54	3.53
M1	L		-39	-16	58	2.60	-39	-19	58	2.02
PPL	L	7	-21	-67	58	6.22	-24	-67	58	3.68
PPL	R	7	39	-49	58	4.66	42	-49	58	2.31
SMA	L/R	6	-3	-1	58	9.99	-3	-1	58	8.83
Putaman	L		-24	-1	6	7.89	-24	2	6	5.69
Putaman	R		24	2	6	5.36	24	2	6	3.56
Thalamus	L		-15	-16	6	4.63	-12	-19	6	3.84
Thalamus	R		15	-16	6	1.82	12	-19	6	0.92
Cerebellum	L		-30	-61	-30	3.91	-30	-61	-30	2.39
Cerebellum	R		30	-58	-30	7.08	30	-58	-30	6.06

Note. MNI coordinates; Abbreviations; PMA-premotor area; M1-primary motor cortex; PPL-posterior parietal lobe; SMA-supplementary motor area; BA-Brodmann’s area.

#### Graph theoretical analysis

Based on the graph theory method, a brain network is a graph composed of edges and nodes; the links between the nodes indicate the functional connectivity between those motor-related brain regions, and the ROIs are denoted by nodes in the graph [27]. The functional connectivity, *η*, between the node i and the node j can be defined as

$$ {\eta}_{ij}={e}^{-\varepsilon {d}_{ij}} $$

where *d*_*ij*_ is the distance between the two nodes, and ξ is a real positive constant. This formula measures how the strength of the relationship between two nodes decreases with the distance between them. A previous study explained that ξ is a subjective selection, and based on the results of previous studies, ξ was here fixed as 2. This particular ξ value allows for reasonable conclusions based on this formula because the connectivity degree supplied by any first-order relationship is *e*^− 2^ ≈ 0.135, which indicates an increase of approximately 13% in the information associated with each particular isolated vertex [47]. Furthermore, *d*_*ij*_ is the distance between the two nodes, calculated as a hyperbolic correlation measure [48]. This calculation is as follows:

$$ {d}_{ij}=\left(1-{c}_{ij}\right)/\left(1+{c}_{ij}\right) $$

Considering the influence of a stimulus-locked response in the task state, in our study, *c*_*ij*_ represents the partial correlation coefficient of two averaged time series,

$$ {c}_{ij}=\left({r}_{ij}-{r}_{i0}{r}_{j0}\right)/\sqrt{\left(1-{r_{i0}}^2\right)\left(1-{r_{j0}}^2\right)} $$

where *r*_*ij*_ represents the Pearson correlation coefficient between the two average time series of node i and node j (i.e., cross-correlating the two averaged time series above). *r*_*i*0_ is the Pearson correlation coefficient between the two time series of node i and the reference function, which in present study, is modeled by the stimulus presentation paradigm.

In this way, the total connectivity degree, *Γ*_*i*_, of a node i in a graph can be defined as the sum of all the connectivity edges between i and all other nodes,

$$ {\varGamma}_i={\displaystyle \sum_{j=1}^n{\eta}_{ij}} $$

This equation describes the amount of information node i is receiving from the other nodes in a particular brain network. In the present study, a larger Γ means that a region is more functionally connected to other regions in the network. Obviously, the Γ takes into account the n-to-1 connectivity using 1-to-1 connectivity measures instead of conventional pairwise connectivity measures [49]. Thus, it is possible to determine changes in the total connectivity degree for some regions by detecting Γ in different brain activity states.

In this study, as there are different time points and different pre-processing times between the pre-test training and post-test training, we normalized *Γ*_*i*_ for node i as follows:

$$ {\overline{\varGamma}}_i={\varGamma}_i/{\displaystyle \sum_{j=1}^n{\varGamma}_i} $$

For the ANOVA model for *η* between the PMA and other ROIs the main factors used were training (pre-test and post-test; within-subjects) and group (experimental group and control group; between-subjects). First, the interaction effect of the *η* between the PMA and the other ROIs was examined. Then, differences between the pre- and post-test for each group and differences between the pre-tests for the two groups were determined. These differences were corrected with the Bonferroni method within the analysis model for each ROI. For each node i, the *η* between the PMA and other ROIs was analyzed statistically by a paired *t*-test between pre- and post-imagination tasks and was further corrected with the Bonferroni method. Moreover, the functional connectivity between one node and the other ROIs is a measurement of pair-wise connectivity, which ignores the changes relative to the total connectivity of all the ROIs. Thus, for the functional connectivity between two nodes, further exploration of the nodes that were significantly altered in *Γ*_*i*_ was carried out. *Γ*_*i*_ is a measurement of the connectivity degree of node i among multiple nodes; namely, it measures the total functional connectivity between node i and all the other nodes.

## Results

**Figure 2:**
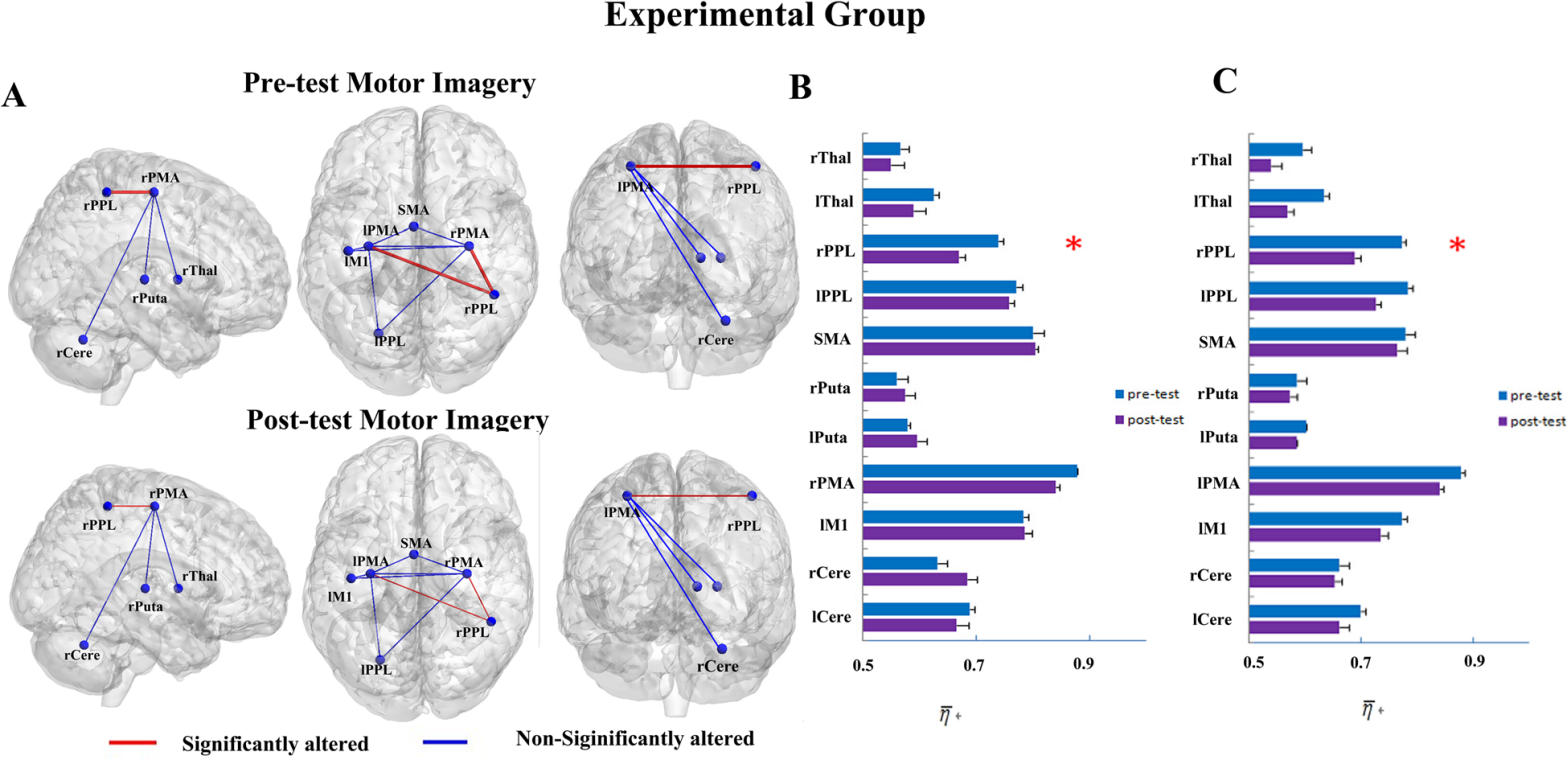
The functional connectivity, *η*, between the lPMA/rPMA and other ROIs of pre-tests and post-tests in imagination task of the experimental group. **(A)** The surface visualization of all 12 ROIs. Red indicates *η* was significantly altered after motor imagery training, while blue indicates *η* was not significantly altered after motor imagery training. **(B)** The *η* of pre-tests and post-tests between the lPMA and other ROIs (* represents significant alterations, corrected p < 0.01). **(C)** The *η* of pre-tests and post-tests between the rPMA and other ROIs (* represents significant alterations, corrected p < 0.005).

Based on our hypothesis, the alteration of *η* between the bilateral PMA and other brain regions engaged in imagination tasks (after regulating for the activities of the rPMA) was analyzed with graph theory. After the motor imagery training, in the experimental group, the *η* between the left PMA (lPMA) and rPPL (Figure 2A and 2B, T(11) = 4.047, corrected p < 0.01) and between the rPMA and rPPL (Figure 2A and C, T(11) = 4.756, corrected p < 0.005) were attenuated at a significant level and were consistent across most subjects (11/12) at the individual level (seen details in Additional file 2: Figure S2). The *η* of the lPMA and other ROIs also showed trends toward alteration after the real-time fMRI motor imagery training for the experimental group, though those changes were not significant. Increasing trends in *η* were detected in the SMA, bilateral Putaman, left M1 (lM1) and left cerebellum (lCere), and decreasing trends in *η* were detected in the bilateral thalamus, left PPL (lPPL), rPMA and right cerebellum (rCere) (Figure 2B). However, the *η* of the rPMA and other ROIs decreased after the real-time fMRI motor imagery training for the experimental group (Figure 2C). Furthermore, there was no significant difference between the experimental and control groups between the bilateral PMA and rPPL at pre-test (baseline condition) (F = 0.782,p > 0.05) during the real-time fMRI motor imagery training.

**Figure 3:**
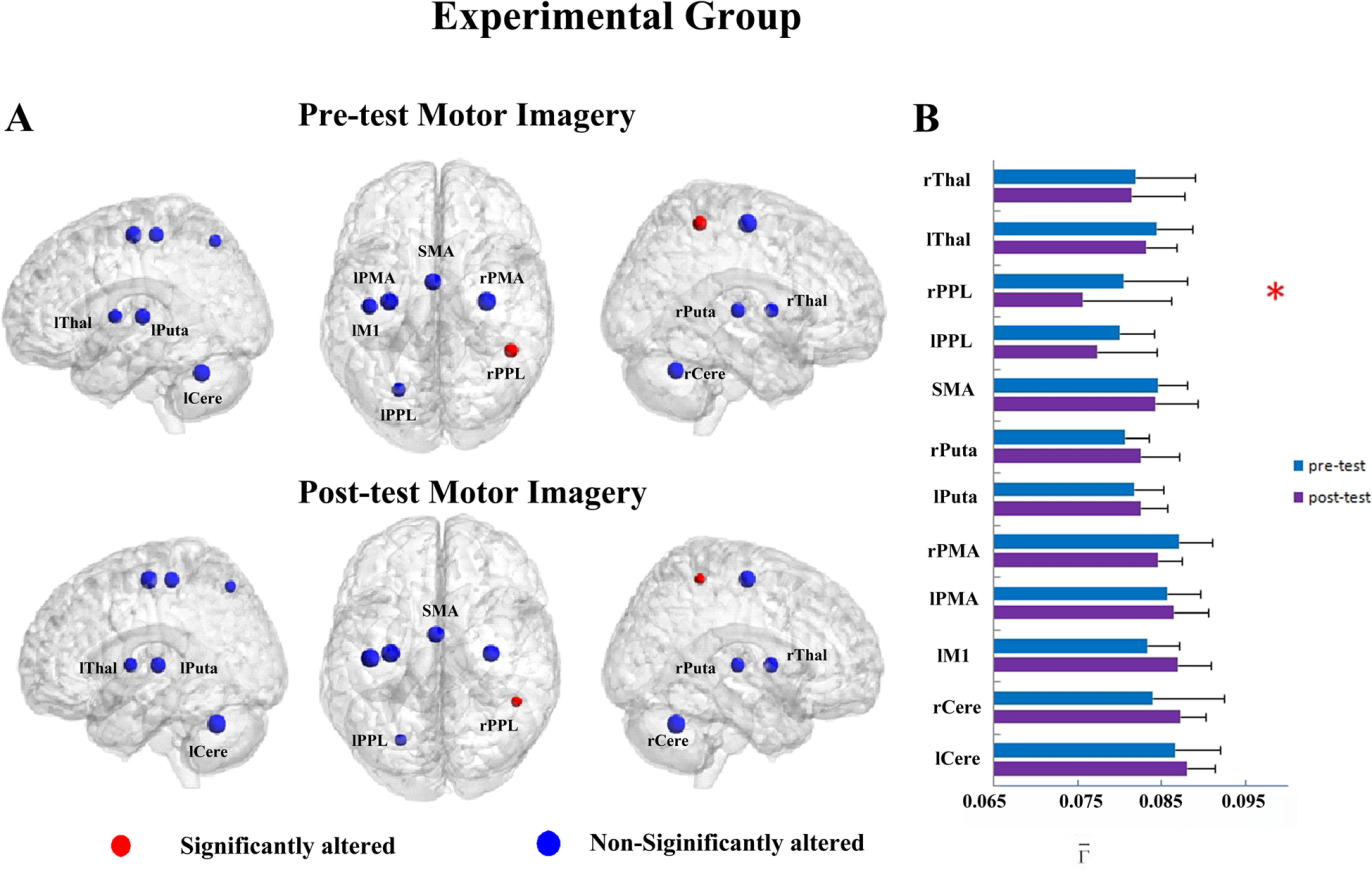
The total connectivity degree, ${\overline{\varGamma}}_i$, of pre-tests and post-tests for all ROIs for imagination task in the experimental group. **(A)** The surface visualization of all 12 ROIs with node sizes indicating the relative value of ${\overline{\varGamma}}_i$. Red indicates that ${\overline{\varGamma}}_i$ of the ROI was significantly altered after motor imagery training, while blue indicates that ${\overline{\varGamma}}_i$of the ROI was not significantly altered after motor imagery training. **(B)** ${\overline{\varGamma}}_i$ of pre-tests and post-tests for all ROIs (* represents significant alterations, corrected p < 0.05).

Moreover, the *η* between the PMA and the other ROIs is a measurement of pair-wise connectivity, which ignores the changes relative to the total connectivity of all the ROIs. Thus, the total connectivity degree, $\overline{\varGamma}$, was further analyzed. A significant interaction effect between learning and group was found in the rPPL (F = 5.890, p < 0.05). In the experimental group, a significant (F = 7.349, corrected p < 0.01) decrease in the $\overline{\varGamma}$ was found for the rPPL for the experimental group (Figure 3A and B), but not for the control group. This decrease in $\overline{\varGamma}$ was observed for each subject, indicating a consistency of alteration across all subjects (see S2). These decreases in total connectivity degree indicate a potential functional dissociation between the rPPL and bilateral PMA for imagination tasks after training. Other ROIs also showed trends toward alteration after the real-time fMRI motor imagery training for the experimental group, though those changes were not significant. Trends toward increases in $\overline{\varGamma}$ occurred in the ROIs of the bilateral Putaman, lM1, lPMA and bilateral cerebellum, and trends toward decreases in $\overline{\varGamma}$ were detected in the SMA, bilateral PPL, rPMA and bilateral thalamus (Figure 3B). Furthermore, there was no significant difference between the experimental and control groups for the rPPL at pre-test (baseline condition) (F = 0.698, p > 0.05) during the real-time fMRI motor imagery training.

## Discussion

Using graph theory, the present study investigated alterations in functional connectivity between PMA and other ROIs during an imagination task. These alterations were induced by self-regulation of the activity of the rPMA. Our results verified the hypothesis that the functional connectivity between the PMA and other brain regions within the motor network when participants are engaged in an imagination task are changed by real-time fMRI motor imagery training.

These significant changes between the bilateral PMA and rPPL might be due to the dominant role of the PMA over other regions of the brain involved in motor sequence training. Another study indicated that the PPL is a critical region of the motor system [50]. Thus, the PPL may also integrate sensory-motor information from other brain regions to generate internal movement images, and such information could be processed by the PMA to formulate a motor plan for motor actions. Furthermore, anatomical connections have been found between the PMA and PPL in macaques [51]. The storage of an acquired skill in the PMA has to be interpreted with the anatomical connections from the PMA to the parietal cortex [52-54]. Thus, we inferred that the PMA and PPL work together to transform and integrate spatial-motor information. Moreover, the *η* between the rPMA and other ROIs were decreased after real-time fMRI training, but the *η* between the lPMA and some other ROIs had increasing trends. This may be due to the greater influence of real-time fMRI training on the rPMA than on the lPMA, and this requires further examination in the future. Due to increased mastery of the skill after motor imagery training, the imagery of motor sequence tapping provided to the experimental group was less dependent on the interaction between the PMA and rPPL compared to the control group. According to the results described above, the functional connectivity between the bilateral PMA and rPPL decreased after motor imagery training; thus, the function related to this alteration may also have been changed.

These significant changes in the total connectivity degree of the rPPL might be attributed to the dominant role of the rPPL in motor sequence training. According to previous studies, the PPL plays important roles in receiving and analyzing somatosensory information in the early stage of training and the memorization of skills in the later stage of training [55-58]. In general, the motor schema was an established process from novelty to automaticity with motor learning [59,60]. At the novelty stage, sensory-motor information was processed by several brain regions, such as the SMA, PMA, M1 and putaman [61,62]. Such information was further integrated in the PPL to generate internal movement images and encode the spatial location of movement as the motor schema [63,64]. In contrast to the sham neurofeedback, true neurofeedback contributes to easier and faster establishment of the motor schema. The movement was gradually automated with this process. After motor imagery training, the motor schema was established, and then, the rPPL may play a role in storing and retrieving the motor schema [65,66]. Thus, the decrease in total connectivity degree of the rPPL was probably due to the established motor schema [65]. Nevertheless, the bilateral PMA and rPPL might play a critical role in the formation of motor planning during imagination tasks.

Overall, the present study demonstrated that the functional connectivity among motor-related regions could be changed by real-time fMRI training. More importantly, such alterations only occurred in the experimental group, which indicated that motor imagery training with true neurofeedback is more effective in altering functional connectivity and total connectivity degree than sham neurofeedback. Exploration of functional connectivity between the PMA and other ROIs revealed that significant alterations induced by motor imagery training occurred in the PMA, which might be attributed to the regulation of the target region (rPMA) or the tight interactions between the PMA and rPPL. Zhang et al. concluded that the motor performance such as tapping speed could be improved and connectivity degree of rPPL was attenuated through half-month offline motor imagery training. However, after the half hour real-time fMRI motor imagery training, the connectivity degree of rPPL could be attenuated [11,46]. That is to say, real-time fMRI motor imagery training might help participants find the strategy quickly to improve their behavior performance by feedback signal of target regions. In any case, the results of the present study are helpful for understanding the alteration of function of motor-related networks after real-time fMRI motor imagery training. Motor imagery training is widely used in sport to improve performance, which raises the possibility of applying it both as a rehabilitation method and to access the motor network independently of recovery. Motor imagery represents an intriguing new “backdoor” approach to accessing the motor system and rehabilitation at all stages of stroke recovery. Unlike active and passive motor therapies, motor imagery, in principle, is not dependent on residual function but still incorporates voluntary drive. In patients with stroke, motor imagery training may therefore provide a substitute for executed movement as a means to activate the motor network [2]. In present study, the functional connectivity between two important brain regions (PMA and rPPL) in the movement network was attenuated after real-time fMRI motor imagery training. According to previous study, PPL is a critical in the parietal-premotor circuit which was suggested to contain the learned contents [67,68], the activities in the PMA were highly correlated with motor performance and the improved motor performance relied more heavily on the functions of PMA [15]. Therefore, these results might be helpful for movement function rehabilitation. Clinically, motor function impairment is a major feature of many neurologic and psychiatric disorders. Therefore, altered functional connectivity induced by self-regulation of the functional activity of the rPMA appears to be promising for clinical application.

## Conclusion

The present study demonstrated that the functional connectivity between PMA and motor-related regions could be changed by real-time fMRI training, and concluded that real-time fMRI motor imagery training might help participants find the strategy quickly to improve their behavior performance by feedback signal of target regions. In any case, the results of the present study are helpful for understanding the alteration of function of motor-related networks after real-time fMRI motor imagery training. Overall, this finding is new evidence that real-time fMRI is effective and can provide a theoretical guidance for the alteration of the motor function of brain regions associated with motor imagery training.

### Limitation

There were several limitations in the current study. Our research, as an exploratory investigation, was more focused on the intrinsic task-related connectivity for before/after motor imagery learning. Thus, graph theory was improved by removing the stimulus-locked response according to the previous study [34]. These removed responses was correlated with the stimulus presentation paradigm, and therefore some worthy results in functional connectivity might be missed by doing so, if the task-related functional connectivity possesses computational correlation with the stimulus presentation paradigm. Moreover, the present experiment only consisted of the real feedback and sham feedback because of the limitation of experiment condition. In future studies, we would add a real control group (receiving no feedback) to provide further convincing results besides the present study. In any case, real-time fMRI motor imagery learning, as an important part of motor learning, is worthy of further investigations at different levels.

## Additional files

Additional file 1: Figure S1. Significant alteration of total connectivity degree, $\overline{\varGamma}$, and functional connectivity, *η*, for each subject during imagination task. (A) The *η* between the bilateral PMA and rPPL for each subject of experimental group. (B) $\overline{\varGamma}$of rPPL for each subject of the experimental group. Additional file 2: Figure S2. Significant alteration of total connectivity degree, $\overline{\varGamma}$, and functional connectivity, *η*, for each subject during imagination task. (A) The *η* between the bilateral PMA and rPPL for each subject of experimental group. (B) $\overline{\varGamma}$of rPPL for each subject of the experimental group.
