# Supplementary material for: Functional connectivity alteration after real-time fMRI motor imagery training through self-regulation of activities of the right premotor cortex
Source: BMC Neurosci. 2015 May 1;16:29. doi: 10.1186/s12868-015-0167-1 (PMC4453277; doi:10.1186/s12868-015-0167-1)
Supplement: Additional file 2: Figure S2. — Significant alteration of total connectivity degree, $\overline{\varGamma}$Γ¯, and functional connectivity, η, for each subject during imagination task. (A) The η between the bilateral PMA and rPPL for each subject of experimental group. (B) $\overline{\varGamma}$Γ¯of rPPL for each subject of the experimental group. [file 12868_2015_167_MOESM2_ESM.docx]

Additional file 2


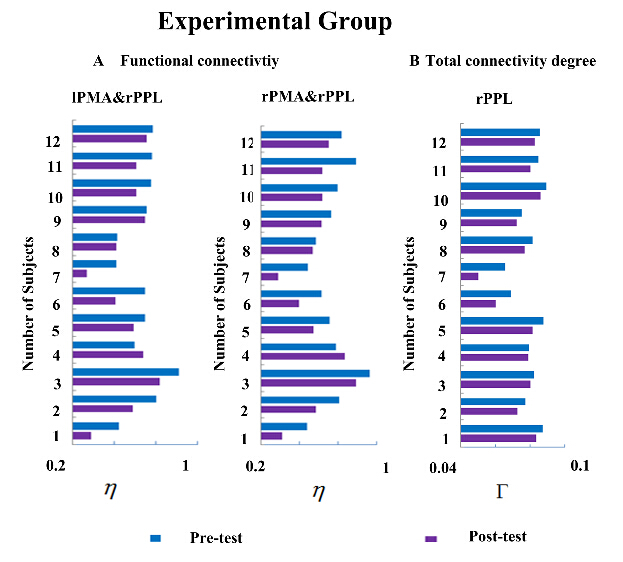


**Figure S2 Significant alteration of total connectivity degree, $\bar{\Gamma}$, and functional connectivity, $\eta$, for each subject during imagination task.** (A) The $\eta$ between the bilateral PMA and rPPL for each subject of experimental group. (B) $\bar{\Gamma}$of rPPL for each subject of the experimental group.
